# Molecular Landscape in Infant High-Grade Gliomas: A Single Center Experience

**DOI:** 10.3390/diagnostics12020372

**Published:** 2022-02-01

**Authors:** Valentina Di Ruscio, Andrea Carai, Giada Del Baldo, Maria Vinci, Antonella Cacchione, Evelina Miele, Sabrina Rossi, Manila Antonelli, Sabina Barresi, Massimo Caulo, Giovanna Stefania Colafati, Angela Mastronuzzi

**Affiliations:** 1 Department of Onco-Hematology, Cell and Gene Therapy, Bambino Gesù Children’s Hospital, Scientific Institute for Reasearch, Hospitalization and Healthcare (IRCCS), 00165 Rome, Italy; valentina.diruscio@opbg.net (V.D.R.); giada.delbaldo@opbg.net (G.D.B.); maria.vinci@opbg.net (M.V.); antonella.cacchione@opbg.net (A.C.); evelina.miele@opbg.net (E.M.); angela.mastronuzzi@opbg.net (A.M.); 2 Neurosurgery Unit, Department of Neurosciences, Bambino Gesù Children’s Hospital, IRCCS, 00165 Rome, Italy; 3 Department of Pathology, Bambino Gesù Children’s Hospital, IRCCS, 00165 Rome, Italy; sabrina2.rossi@opbg.net (S.R.); sabina.barresi@opbg.net (S.B.); 4 Department of Radiological, Oncological and Anatomo-Pathological Sciences, University Sapienza of Rome, 00185 Rome, Italy; manila.antonelli@uniroma1.it; 5 Department of Neuroscience, Imaging and Clinical Sciences, G. D’Annunzio University of Chieti, 66100 Chieti, Italy; massimo.caulo@unich.it; 6 Department of Diagnostic Imaging Oncological Neuroradiology Unit, Bambino Gesù Children’s Hospital, IRCCS, 00165 Rome, Italy; gstefania.colafati@opbg.net

**Keywords:** pediatric high-grade gliomas, infants, molecular profile, oncogenic fusions, target therapy, larotrectinib

## Abstract

High-grade gliomas (HGG) represent about 15% of all pediatric brain tumors, with a dismal prognosis and survival rates ranging from 15 to 35%. Approximately 10–12% of pediatric HGGs (pHGG) occur in children younger than five years of age at diagnosis, specifically infants (iHGG), with an unexpected overall survival rate (OS) in 60–70% of cases. In the literature, iHGGs include a large variety of heterogeneous lesions with different molecular profiles that likely explain their different outcomes. We report our single-institution experience of iHGG including 11 children under five years of age with newly diagnosed HGG between 2011 and 2021. All patients received surgery and adjuvant chemotherapy; only two patients received radiotherapy because their age at diagnosis was more than four years-old. Molecular investigations, including next generation sequencing (NGS) and DNA methylation, detected three NTRK-fusions, one ROS1-fusions, one MN1-rearrangement, and two PATZ1-fusions. According to the molecular results, when chemotherapy failed to control the disease, two patients benefited from target therapy with a NTRK-Inhibitor larotrectinib, achieving a complete remission and a very good partial response, respectively, and no severe side-effects. In conclusion, molecular investigations play a fundamental role in the diagnostic work-up and also in the therapeutic decision. Their routine use in clinical practice could help to replace highly toxic chemotherapy regimens with a target therapy that has moderate adverse effects, even in long-term follow-up.

## 1. Introduction

Pediatric high-grade gliomas (pHGG) represent approximately 15% of all central nervous system (CNS) tumors in children [1].

Rarely, pHGG develops in infants, with an age at diagnosis ranging from three to five years-old [2], typically occurring in the cerebral hemispheres and less frequently in the posterior fossa or other midline locations [3]. Currently, there is no universally accepted treatment protocol for patients with pHGG, and infant population is not an exception. Available options include surgery [4], chemotherapy [5], radiation [6], and the recently available targeted therapies [7].

Surgical treatment has a non-negligible functional and mortality risk in infants due to the considerable size of the tumor at the time of presentation and the limited circulating blood volume. Gross total resection (GTR) is usually difficult to obtain due to both the infiltrative growth pattern of these tumors and the necessity to preserve neurological functions [8].

In the early 1980s, standard treatment included surgery and radiotherapy [9] before the development of chemotherapeutic regimens that could have been applied to this vulnerable group [10]. Infants diagnosed with HGG do not usually receive primary radiotherapy unlike older pediatric and adult patients, in order to avoid unacceptable neurological sequelae. Instead, they often receive chemotherapy as a first-line approach, after primary neurosurgical resection. Currently, there is no standardized treatment protocol for pHGG, apart from several studies that have suggested a combination of multiple drugs (including methotrexate, etoposide, carboplatin or cisplatin) as a valid approach [11], as well as high-dose regimens, taking advantage of dose-dependent brain tumor sensitivity to chemotherapy [5].

However, the prognosis of pHGG remains poor, with a 5-year overall survival (OS) rate of 20% for children younger than 14 years [12]. Nevertheless, long-term OS is usually more favorable in infants when compared with older age groups, ranging between 60 and 70% at 10-years follow-up, even with incomplete resection and without irradiation [13].

These results are likely due to specific molecular profiles and different tumor biology reported across age groups [14].

Histological diagnosis and grading are of undisputable value for the diagnosis of HGG, but molecular investigations have recently confirmed the fundamental role of epigenetic features to achieve the correct diagnosis. Nonetheless, the 2021 version of the World Health Organization (WHO) classification of CNS tumors includes molecular parameters as biomarkers of grading with an intrinsic prognostic value [15].

Infant HGG (iHGG) molecularly differs from adult and pHGG: in fact, even in the presence of common histological and immunohistochemical features, iHGG harbors specific genetic and epigenetic alterations [16]. Molecular alterations usually detected in adult glioblastoma such as overexpression of EGFR and PDGFR as well as TP53, PTEN, and BRAF V600E mutations are rare in infants [17].

Conversely, there is evidence demonstrating that iHGG harbors mutations or fusions on the ALK, NTRK1/2/3, ROS1, and MET genes [2]. The peculiar molecular landscape of iHGG has prompted the introduction in the 2021 WHO classification of CNS tumors of a new entity named “*Infant-type hemispheric glioma*”, which typically occurs in newborns and infants [18].

In this study, we present a series of iHGG referred to our hospital in the last 10 years, detailing the histological characteristics, molecular and epigenetic profiles, treatment, and outcome.

## 2. Materials and Methods

### 2.1. Population

All patients who presented from January 2011 to January 2021 at Bambino Gesù Children’s Hospital with histologically confirmed HGG were considered for this study.

Children with H3K27-altered tumors were not included in this series.

### 2.2. Imaging

MRI was performed with 1.5 Tesla (Siemens Magnetom Vision Plus, Erlangen, Germany) and 3 Tesla (Siemens Magnetom Skyra, Erlangen, Germany) scanners using a standardized pediatric tumor protocol including multiplanar T1- and T2-weighted, FLAIR (fluid-attenuated inversion recovery), DWI sequences, and post-contrast T1-weighted sequences. The section thickness for acquired 2D imaging sequences was 4 mm on the 1.5 T magnet and 3 mm on the 3 T magnet with a minimal gap or no gap. All patients were unable to cooperate for age-related conditions and they were imaged under general anesthesia.

All patients received a scan of the entire neuraxis at diagnosis and during follow-up. The extent of tumor resection was quantified based on an early postoperative (within 48–72 h) MR study. Radiological examinations were performed during treatment according to protocol or every three months after any therapy modification. Additional MRI were carried out according to the patient’s clinical conditions (worsening or development of new neurological symptoms).

MR studies were retrospectively reviewed by two blinded experienced pediatric neuroradiologists (MC and GSC, 23 years of experience), in accordance with the most recent Response Assessment in Pediatric Neuro-Oncology (RAPNO) criteria [19].

### 2.3. Molecular and Epigenetic Investigations

Histological diagnosis was performed by two independent experienced neuro-pathologists for all cases.

Molecular investigations included next generation sequence (NGS) studies on RNA samples and DNA methylation profiling.

RNA was extracted from fresh frozen or formalin-fixed tissue. All libraries were sequenced using Illumina HiSeq (Illumina, San Diego, CA, USA). Data analysis was then performed using Archer FusionPlex OPBG custom software or, alternatively, Sanger sequencing. NGS was performed on RNA samples, investigating a fusion panel consisting of 52 genes, usually associated with pediatric brain tumors (such as ALK, BRAF, BRD3, BRD4, CAMTA1, CCNB3, CIC, EGFR, EPC1, ERG, ETV6, EWSR1, FGFR1, FGFR2, FGFR3, FOXO1, FUS, GLI1, HMGA2, MAML2, MET, MYB, MYBL1, MN1, YAP1, NCOA2, NOTCH1, NOTCH2, NTRK1, NTRK2, NTRK3, NUTM1, PDGFB, PDGFRA, PDGFRB, PIK3CA, PLAG1, PRKCA, RAF1, RELA, RET, ROS1, SS18, STAT6, TAF15, TERT, TFE3, TFEB, TFG, USP6, YWHAE, and VGGL2).

DNA methylation profiling was performed on tissue samples with the highest tumor cell content (usually more than 70%) using the Infinium Methylation EPIC BeadChip array (850k), according to the manufacturer’s specifications (Illumina, San Diego, CA, USA). Methylation data were subsequently uploaded to the Heidelberg Brain Tumor classifier (version v11b4) and compared to the reference cohort previously profiled at the German Research Center (DKFZ). Classification results were then reported and integrated by an expert neuropathologist according to the clinical and histological findings for each patient. Recently, they were critically reviewed according to the WHO classification of CNS tumors with the new classifier v12.3.

### 2.4. Statistical Analysis

Progression free survival (PFS) was defined as the time from the surgery leading to the initial diagnosis to the first radiological progression. OS was defined from date of diagnosis to date of death or last available follow-up. Given the small sample size, no further statistical investigations were performed.

## 3. Results

### 3.1. Population

Between January 2011 and January 2021, 64 pediatric patients with histologically confirmed HGG without the H3K27m mutation were referred to the Bambino Gesù Children’s Hospital. This group included 11 children younger than five years of age at diagnosis (17%). The most frequent location was at cerebral hemispheres in 8/11 (72%), spinal cord in 2/11 (18%), and at the cerebellopontine angle in 1/11 patient (10%). Metastatic disease was ruled out in all cases by cytology on cerebrospinal fluid (CSF) lumbar tapping and brain and spinal contrast-enhanced MRI at diagnosis. In our cohort, median age at diagnosis was 22.5 months (range 0–46 months) with a single patient being diagnosed with a congenital HGG (at 36 weeks of gestational age). Eight of 11 patients were female (2%). The population details are reported in Table 1.

All cases were extensively discussed with our multidisciplinary and molecular tumor board, and the goals of surgery were shared among participants. As a general rule, a maximal safe resection was always considered.

A GTR was obtained in six infants (54.5%), a subtotal resection (STR) being the best achievable result in the remaining five patients (45.5%). None of the patients exhibited major neurological complications, and all had a Lansky performance status above 60 after surgery, allowing for subsequent adjuvant treatment.

### 3.2. Histological, Molecular and Epigenetic Results

Tissue samples were diagnostic and adequate for histological diagnosis in all cases.

Histology showed 9/11 HGG (81%), 1/11 high-grade glioneuronal tumor (HGGNT) (9%), and 1/11 high-grade neuroepithelial tumor (HGNET) (9%).

Additional molecular analysis was performed in all cases. In two cases, the available histological samples were inadequate to perform molecular investigations.

NGS-RNA was performed in 9/11 patients and revealed three tumors with NTRK-fusions, two with PATZ1-fusion and one with ROS1-fusion.

DNA methylation was performed in 9/11 patients. In six cases, the following matches were highlighted: one iHGG, two IDH wild-type, one BCOR internal tandem duplication (BCOR-ITD), one plexus tumor, and one MN1-rearrangement. In this last case, RNA sequencing did not show any detectable fusion genes in the panel, but DNA methylation demonstrated a MN1-rearrangement, helping to achieve the correct diagnosis.

In three cases, no match was detected when comparing the results with the reference cohort.

With the new classifier version (v12.3) all nine patients were critically re-investigated and the re-examination revealed a match with the reference cohort in all cases. In particular, this revaluation confirmed the one iHGG, one with NTRK-fusion, one BCOR-ITD, and the MN1-rearranged neoplasm already identified. The ROS1-fused neoplasm, which previously matched the IDH wild-type subclass mesenchymal, was found coupled with anaplastic pleomorphic xantoastrocyoma (PXA). A previous IDH wild-type subclass midline was reinterpreted as a pHGG RTK1 type, subtype B.

Impressively, in the three previously not matched-cases, the re-analyzation showed two tumors with PATZ1-fusions and one pHGG RTK1 type, subtype A, confirming previous molecular information first detected by NGS-RNA.

Details of the molecular investigations are reported in Table 1.

### 3.3. Treatment

Nine of 11 patients (81%) received adjuvant chemotherapy, according to the Associazione Italiana di Ematologia e Oncologia Pediatrica (AIEOP) PNET infant indications. After three courses of induction chemotherapy (methotrexate 8 g/m^2^ plus vincristine 1.5 mg/m^2^ week 0; etoposide 2.4 g/m^2^ week 1; cyclophosphamide 4 g/m^2^ plus vincristine 1.5 mg/m^2^ week 4), patients underwent two courses of high-dose thiotepa (300 mg/m^2^ for three days, week 7) followed by autologous hematopoietic stem cell transplantation [20]. No radiation was proposed in light of the children’s age. Two of nine patients respectively experienced progressive and refractory disease after the induction chemotherapy; therefore, they did not receive HD-CT (high-dose chemotherapy), and, according to molecular detection of an NTRK-fusion, both started a target therapy with the NTRK-inhibitor larotrectinib.

Two patients, older than four years-old at the time of diagnosis, received focal radiation (54 Gy) and adjuvant chemotherapy with temozolomide [21].

First and second-line treatments are described in Table 2.

### 3.4. Progressive and Relapsed Disease

Three patients with three hemispheric HGG, experienced a disease progression with a median time to progress of 47.5 months from diagnosis (range 3–91 months). One patient (patient no 4) with a spinal HGG experienced a disease refractory after primary induction. 

Three patients, two hemispheric and one posterior fossa HGG, developed a disease relapse, with a median time to relapse of 3.6 months from diagnosis (range 3–5 months). Four of these seven relapsed/progressive/refractory patients underwent a second neurosurgical approach; conversely, in 2/7 patients, surgery was not advised and second-line treatment was started.

Second-line regimens were administered in 5/7 patients with relapsed or progressive disease. In 2/7 patients, a rapid progressive disease developed after standard chemotherapy, before any second-line regimens could be explored.

In three cases of hemispheric HGG (patients 4, 8, and 11), an anti-angiogenetic inhibitor (bevacizumab 10 mg/kg intravenously every two weeks) was administered, associated with a topoisomerase inhibitor (irinotecan 125 mg/m^2^ intravenously every two weeks). This treatment was started after focal radiotherapy in 2/3 cases.

Patient 8, in particular, with a hemispheric HGG, received a second GTR and an adjuvant chemotherapy combination with two antineoplastic agents, bevacizumab and irinotecan (12 cycles) following local radiotherapy. She achieved a complete disease remission, which was maintained at 10-years follow-up. NTRK-fusion was identified *a posteriori*, because at the time of diagnosis (March 2011), molecular investigations were not routinely carried out, and the target therapy was not an available option.

Patient 1, with a congenital HGG, demonstrated progression of disease after four cycles of chemotherapy, and started target therapy first with crizotinib and then with larotrectinib, subsequently achieving complete remission at 2-years follow-up, in the absence of treatment-related severe adverse events [2].

Patient 7 was a two-year-old girl with a NTRK-fused spinal HGG, who showed a refractory disease after four cycles of standard chemotherapy: she started target therapy with larotrectinib and, after the 4-month follow-up, she achieved a partial (<50%) reduction in the volume of pathological tissue of the dorsal spine.

**Figure 1:**
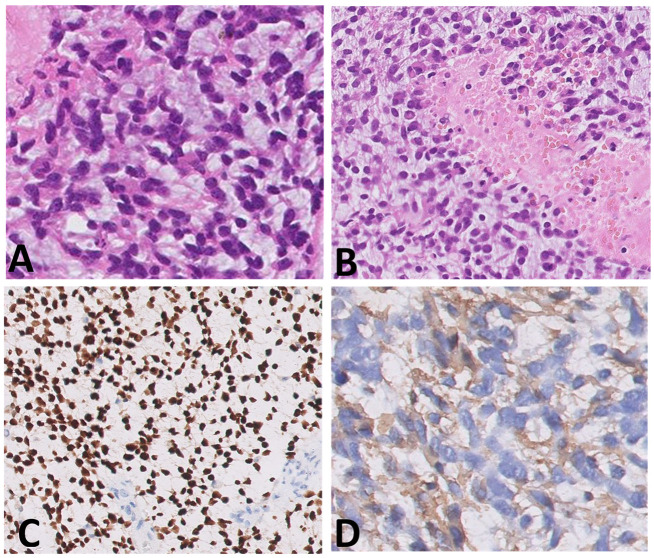

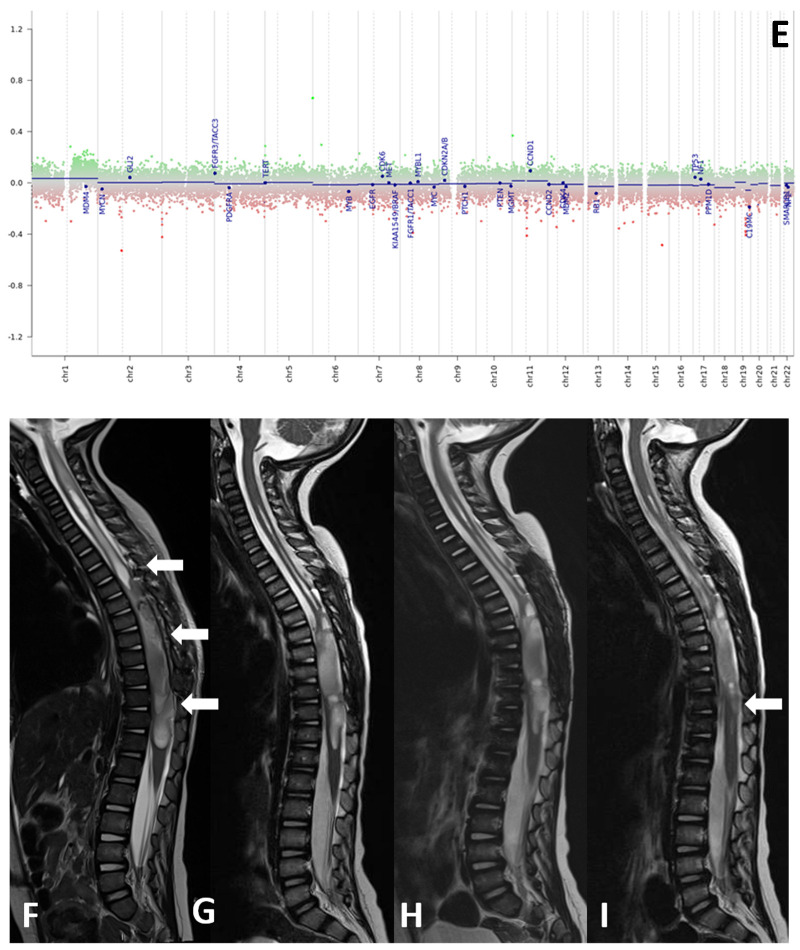
(**A**–**I**) Histological samples (**A**–**D**), molecular profile (**E**), and MRI imaging at diagnosis (**F**), after standard chemotherapy regimens (**G**) and at three months (**H**) and six months (**I**) after the start of larotrectnib in patient number 7. The tumor consisted of small spindle cells embedded in a myxoid background (**A**). Mitoses were present throughout. The tumor showed foci of necrosis (**B**) and microvascular proliferation. OLIG2 was diffusely expressed (**C**); GFAP expression was multifocal (**D**). Molecular profiling with classifier v11.b4 did not reveal any match. Revaluation with classifier v12.3 showed GBM pedRTK1a glioblastoma, pediatric RTK1 type, subtype A (score 0.31337). At imaging evaluation, the neoplasm is characterized by solid-cystic aspect. Sagittal T2-weighted sequences at four consecutive time points: post first surgery approach (arrows, (**F**)), after induction chemotherapy (**G**), after three months (**H**) and after six months after the start of larotrectnib (**I**). At the last follow-up, there was a moderate reduction in the size of the neoplasm, especially in the solid caudal portions (arrow, (**I**)).

Histological samples, molecular profiles, and MRI imaging of this patient are reported in Figure 1.

In our cohort, 4/11 patients died of progressive disease (36% of the study population) including one patient with BCOR-ITD, one pediatric RTK1 type HGG subtype B, and two patients without molecular investigations for inadequate tissue samples.

Median OS was 42 months (range 3–162 months).

Among them, in one case of progressive disease (patient 9), a surgical approach was tempted, achieving a GTR; nonetheless, clinical conditions quickly worsened before a second-line therapy could be explored and patient died of metastatic disease.

The second patient (patient 4) with a hemispheric HGG, IDH wild-type subclass mesenchymal at molecular investigations, received a GTR, followed by radiotherapy and chemotherapy with bevacizumab and irinotecan (12 cycles). She died of metastatic disease 96 months after primary diagnosis. Interestingly, molecular analysis review conducted on tumor samples with the new v12.3 classifier showed a match with pediatric RTK1 type, subtype B, and a complex copy number variation (CNV) with CDK6 and MET amplification, and a platelet-derived growth factor A (PDGFRA) gain.

**Figure 2:**
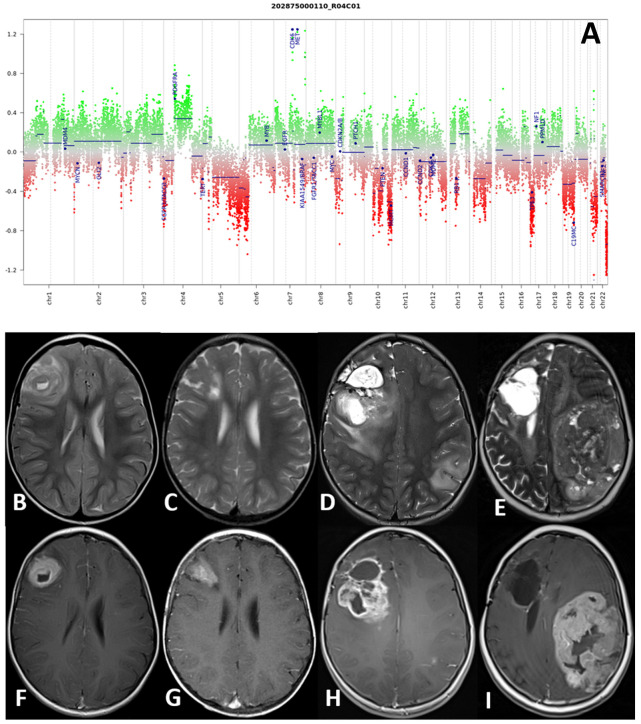
(**A**–**I**) Molecular profile (**A**) and MRI at four consecutive time-points of patient number 4. DNA methylation profile showed a complex copy number variation (CNV) with CDK6 and MET amplification, and a platelet-derived growth factor A (PDGFRA) gain (**A**). Axial TSE T2-weighted (**upper row**) and post-gadolinium SE T1-weighted (**lower row**) sequences at four consecutive time points: at presentation (**B**,**F**), at first local recurrence after surgery (**C**,**G**), at second local recurrence after reoperation (**D**,**H**), and spreading to the contralateral hemisphere (**E**,**I**). The right frontal high-grade glioma and recurrences present with a non-homogeneous hyperintense signal on T2-weighted and intense and non-homogenous contrast enhancement on T1-weighted images.

Histological samples, molecular profile, and MRI imaging of this patient are reported in Figure 2.

The third patient with a hemispheric HGG (patient 11) was treated at diagnosis with a STR and at relapse, she received chemotherapy with bevacizumab and irinotecan (eight cycles). She died after metastatic spread dissemination 33 months after diagnosis.

The fourth patient with a BCOR-ITD HGG of cranial posterior fossa (patient 3) experienced a first local relapse 23 months after initial diagnosis, and a GTR was carried out. Histological and molecular examinations confirmed a primary tumor’s relapse with the same molecular profile. The family initially refused further oncological treatments, and the child was lost to follow-up for almost two years. He then returned to our attention due to progressive neurological worsening. After the third relapse was radiologically documented, the patient experienced another GTR, but no treatment was started due to rapid progressive leptomeningeal dissemination. He died 64 months after primary diagnosis [22].

At last follow-up in January 2021, 7/11 patients were alive (63%). Two of them, with NTRK-fusion tumors, are still in treatment with larotrectinib. Five of seven patients alive (71%) discontinued therapy and are in complete disease remission.

## 4. Discussion

pHGGs usually present as distinct and heterogeneous spectrum of entities, which strongly differ from their adult counterparts. Infant cases represent about 10% of all pediatric CNS tumors, and 5% of them in the first six months of life [14].

Infant patients are the most vulnerable subgroup, both for age related issues and the highest frequency of germ-line mutations in this particular population [23].

Treatment challenges for clinicians include large tumor size at presentation due to delayed onset of symptoms in a high-plastic skull, and a significant radiological and histological heterogeneity, adding complexity to obtain accurate diagnosis [24].

Different studies have reported positive correlations between the extent of resection and improved PFS and OS rates [25]. In another study of a small sample size of 21 infants, the authors reported an improved OS among patients who experienced a GTR compared to those who underwent a STR [10]. However, surgical resection is challenging due to several factors such as the voluminous tumor size at diagnosis, the frequent hemorrhagic nature of tumor tissue, and the small circulating blood volume, with evidence of increased perioperative morbidity and mortality rates [26]. Therefore, a rational use of explorative biopsy and subsequent chemotherapy regimens have been advocated in these cases [7].

At the time of diagnosis, all patients in our cohort received a resective neurosurgical approach, obtaining 5 GTR and 6 STR, without surgery-related short or long-term sequelae.

Radiotherapy is usually avoided in infants due to the well-known detrimental impact on their subsequent psychomotor development [27]. Therefore, the poor prognosis is intricate with a higher incidence of early and late toxicities, experienced by long-term survivors [28]. Ali et al. investigated 132 children with CSN tumors, identifying several factors as predictors of neurocognitive outcome such as lower baseline QI, supratentorial tumor location, and younger age at diagnosis; however, the authors did not report any negative impact of adjuvant treatment (chemotherapy and/or focal RT) [29].

In our cohort, only two patients aged more than four years at diagnosis received focal radiation with concomitant oral temozolomide. At last available follow-up, one patient had died due to progressive disease 33 months after primary diagnosis; the other patient, a hemispheric HGG with ROS1-fusion, discontinued treatment and is in complete disease remission at 12-months follow-up; no neurocognitive impairment has been documented with neurological performance tests.

Currently, histological diagnosis is no longer sufficient to correctly classify and treat CNS pediatric tumors, and HGGs are no exception [2].

Despite a limited repertoire of histologies, we observed a high biological heterogeneity in our cohort, further supporting previous experiences available in the literature. In particular, molecular and epigenetic modifications largely differ between similar histologies. CNS gliomas show one of the most extensive landscapes of molecular and epigenetic alterations, useful to clarify their etiology, to correctly define molecular subtypes with a different biology, and, moreover, to strongly correlate to prognosis [30]. Understanding their peculiar profiles is fundamental in order to predict resistance or refractory to common treatments, and the possible employment of specific target therapies [31].

Several molecular alterations have been identified in hemispheric pHGG including the PI3K/AKT, Ras-Raf-MEK-ERK, RB, and p53 pathways [32,33], and a particular mention concerns infant population. In fact, in contrast to adult gliomas, pediatric, especially iHGG (especially those arising in children younger than 1 year), share a common genetic feature represented by the presence of receptor tyrosine kinase (RTK) alterations, harboring fusions in ALK, ROS1, NTRK1/2/3, and MET genes, reported in almost 61–83% of cases [34].

In a large report of a multi-institutional 150 iHGG cohort, Guerriero et al. identified three subgroups with specific molecular alterations embracing NTRK and RAS/MAPK pathways in both supratentorial and midline HGG, which suggests a complete new identity of iHGG [18]. The 5th edition of the WHO classification of CNS tumors strongly advocates the integration of histology with a molecular diagnostic, recognizing a new identity named “HGG infant-type hemispheric glioma with NTRK alterations” [15].

Beyond the indisputable impact of recent molecular findings for basic science, one of the most intriguing aspects is represented by their potential translation into the clinic. Unveiling specific molecular markers could change the therapeutic paradigm of this disease, historically based on surgery and chemotherapy regimens.

Furthermore, cancer epigenetics, which determine an interruption in gene expression and a consequent dysregulation of signaling pathways and changes in cellular phenotype and behavior, could help to explain unknown resistance patterns, which are involved in refractory to treatment [35] and have significant impact on the prognosis, similar to the canonical risk factor of the extent of resection in the neurosurgical approach [36].

One of the major studies on iHGG was published in 2020 by Clarke et al., who reported a unique series of almost 130 iHGG with an intrinsic peculiar phenotype. In large, they identified targetable MAPK alterations, and in the other cases, driving gene fusions such as ALK, NTRK1/2/3, ROS1, and MET with explicit impact on prognosis [2].

In our study population, we identified the NTRK-alteration, described in about 40% of iHGG [37], in 3/11 patients (27%).

The NTRK receptors are fundamental transducers for a wide spectrum of developmental signals for the neural cells including apoptosis induction, proliferation, and differentiation [38]. All NTRK-fusions involve the C-terminal kinase domain of NTRK1,2,3 with the N-terminal sequences of several different genes, with consequently constitutional activation of the chimeric protein product. The most frequently reported fusion is ETV6–NTRK3, but several case reports in the literature have identified additional mutations such as EML4–NTRK3 and TPM3–NTRK1 fusions [18,38].

NTRK gene fusions are of particular interest because of their potential therapeutic role, with the growing availability of NTRK-inhibitors in recent years [39,40]. A recently published phase 1/2 clinical trial of NTRK positive patients demonstrated an objective response in 74% of included patients (with age ranging from one month to 84 years old), with a complete and prolonged response in about 16% [41]. A phase 1 pediatric trial including 24 patients with NTRK-fused tumors demonstrated that larotrectnib is safe and feasible among the pediatric population, without severe adverse events; furthermore, 93% of patients achieved a complete response after the start of target therapy. Phase 2 is still ongoing [42].

Two patients in our cohort received larotrectinib after completing first line chemotherapy: in one case, larotrectinib was administered after radiological and clinical evidence of progressive disease and induced an impressive complete remission at the 2-year follow-up, without severe side effects or impact on neurological development; the second patient, with refractory disease after induction, received the NTRK-inhibitor with a very good partial response documented in MRI studies at four and six months after starting larotrectinib (Figure 1).

Other alterations involving ALK, ROS1, and MET have been specifically detected in the infant subset [2]. In our cohort, one patient with a hemispheric HGG was detected with a ROS1-fusion.

The proto-oncogene tyrosine kinase ROS1 is an orphan receptor that is normally expressed only during neurological development, structurally similar to the anaplastic lymphoma kinase (ALK). ROS1 fusions are potent oncogenic drivers through a constitutive kinase activation, which activate several signaling pathways of cell differentiation and survival (such as PI3K/AKT/mTOR, JAK/STAT, and MAPK/ERK) [43]. It was initially detected in glioblastoma tumor lines and more recently in lung adenocarcinomas [44,45], thus leading to the Food and Drug Administation (FDA) approval of the small tyrosine kinase inhibitor crizotinib for the treatment of metastatic non-small-cell lung cancer (NSCLC) [46].

The ZCCH8-ROS1 fusion detected in our patient was primarily identified by Wiesner and colleagues in 2014 in a Spitz nevi tumor [47]. More recently, Coccè et al. identified these fusion genes in a congenital glioblastoma in a 2-month-old boy, suggesting that ROS1 rearrangement could act as a key oncogenic driver in a specifical subset of GBM [48], in addition to the well-known GOPC-ROS1 fusion, largely reported in the congenital glioblastoma series [49,50].

Our patient received local radiotherapy with concomitant temozolomide, achieving a complete disease remission at 12-months follow-up. No target therapy was started in this patient, but in the case of relapsed disease, we hypothesize that crizotinib would be a valid option to investigate.

Our molecular investigations also detected two tumors with PATZ1-fusion, which is in line with the literature data, describing them as the most frequent alterations in hemispheric HGG, following NTRK ones [18].

Recent reports have identified a handful of pediatric brain tumors displaying a fusion of the PATZ1 gene with either MN1 or EWSR1 as a partner. The histological diagnosis of these tumors included several histotypes (glial, glioneuronal and polyphenotypic morphologies), with some potential similarities but also significant differences in appearance, which may justify previous recognition as a defined entity [51,52].

The EWSR-PATZ1 fusion detected in one patient in our cohort has been described as a rare rearrangement in small round cell sarcoma and low- and high-grade glioneuronal neoplasms [53]. This detection was particularly important because histological diagnosis had been challenging for non-specific immunostaining panels including neuronal, glial, epithelial, and mesenchymal markers. Molecular investigations, allowing for the detection of specific hallmarks in this poorly differentiated neoplasm, might be determinant in defining these new entities, allowing for speculation on the biological behavior of tumors with identical molecular features arising in different locations [54].

MN1 alteration characterizes a new class of central nervous system high-grade neuroepithelial tumors that have recently been identified based on epigenetic investigations and profiles of a large cohort of tumor samples [55]. Wood et al. identified a MN1-rearranged tumor not assigned to any brain tumor methylation class [56].

The MN1-PATZ1 rearrangement, which has already been reported in the literature, may define a novel histological malignant pediatric brain tumor, which is closely related to PATZ1-sarcomas [52].

In our study, we reported two MN1-rearranged tumors: in one case, NGS investigation detected MN1-PATZ1 fusion, which was also confirmed in methylation profiling; in the other case, molecular diagnostics did not reveal any potential oncogenic driver fusion, but DNA methylation profiling showed a MN1 rearrangement, guiding our final diagnosis.

In one patient, DNA methylation revealed a BCOR-ITD, usually associated with a high-grade neuroepithelial tumor, which has recently been reclassified in 2016, and reconfirmed in the 2021 WHO classification of CNS tumors as a new distinct group, previously belonging to the pediatric neuroectodermal tumor (PNET) family. They presented a characteristic molecular alteration involving the BCOR gene that acts as an inhibitor of the BCL6 gene involved in cellular proliferation and differentiation [55]. They usually affect infants and are associated with a poor prognosis, refractory to therapies, and high tendency to relapse [57].

This patient from our cohort (patient no 3) has been described in a recent case series as one of the few cases already reported in the literature (almost 24 cases worldwide) [22], with, as expected, a higher refractory to treatments and a poor prognosis.

Median OS was in line with the previous literature data, showing a better outcome among iHGG because 7/11 patients (63%) were still alive at last follow-up.

Five of them discontinued treatment and are in complete disease remission.

## 5. Conclusions

Our study reported on a single-institution of a iHGG population diagnosed between 2011 and 2021.

Primarily, in our experience, we suggested that, in order to obtain the best possible treatment for patients, the cooperation of a multidisciplinary team is quite essential, with strong collaboration in the diagnostic and therapeutic pathway of a dedicated working group including pediatric oncologists, neurosurgeons, neuroradiologists, and anatomo-pathologists. Multidisciplinary teams have now become the standard of care for cancer patients, in order to improve diagnosis, treatment, survival, and quality of life [58], even more confrontinq with newly and still unknown tumor entities.

A major objective limitation of our study is surely represented by the small sample size of the population, and further investigations on a larger number of patients are required to confirm our results. Nonetheless, the major available data on larger infant populations first reported by Guerriero et al. [18], and then by Clarke and colleagues [2], strongly demonstrated that iHGG have specific molecular driving alterations, that in fact could impact prognosis. Our results, even if in a small sample, confirmed the data previously reported in the literature. We identified three NTRK fusions and one ROS1 rearrangement, two of the most frequent alterations among iHGG. Meanwhile, we evidenced two MN1 rearranged and one PATZ1 fused tumors, not reported in the larger series above-mentioned, but well-known entities among pediatric brain tumors.

The detection of a wide spectrum of molecular alterations strongly suggests that more efforts need to be taken to increase the knowledge of these tumor entities and their peculiar molecular profiles. Even if in a small population, our results offer support to speculate concerning the potential role of a molecularly driven targeted therapy approach in the challenging scenario of treatment-resistant iHGG. Unsatisfactory outcomes of currently available treatments are well fitted with the exquisite heterogeneity of targetable molecular defects in these tumors. Evidence of clinical tolerability, even in infants, and the absence of mild or severe side-effects might represent an additional aspect supporting the cautious introduction of targeted therapy for this challenging disease, whenever alternative treatment options with similar risk profile are not available or have been demonstrated to be ineffective.

## Figures and Tables

**Table 1:** Characteristics of population.

N°	Age (m)	Site	Diagnosis	NGS-RNA	DNA Methylation Profile (v11.b4)	DNA Methylation Profile (v12.3)	PFS (m)	OS(m)	Status (D/A)
1	0	RH	HGG	ETV6-NTRK3	IHG (score 0.99)	IHG (score 0.99)	5	47	A
2	20	RH	HGGNT	EWSR1-PATZ1	NM	HGGNT-PATZ1 (score 1)	NA	18	A
3	15	PCF	HGG	NM	HGNET BCOR-ITD (score 0.9)	HGNET BCOR-ITD (HGNET-BCOR) (score 0.99)	31	64	D
4	19	RH	HGG	NM	GBM IDH WT, subclass midline (score 0.61)	GBM, pediatric RTK1 type subtype B (score 0.44)	3	96	D
5	20	RH	HGNET	MN1-PATZ1	NM	HGNET-PATZ1 (score 0.99)	NA	3	A
6	30	SC	HGG	NM	HGNET MN1 alteration (score 0.99)	HGNET-MN1 (score 0.99)	NA	3	A
7	13	SC	HGG	MEF2D-NTRK1	NM	GBM pediatric RTK1 type subtype A (score 0.31337)	NA	8	A
8	30	LH	HGG	KCTD8-NTRK2	Plexus tumor, subclass pediatric B (score 0.4)	GBM pediatric RTK1 type, subtype B (score 0.43)	22	162	A
9	17	LH	HGG	NA	NA	NA	3	6	D
10	38	RH	HGG	ZCCH8C-ROS1	GBM IDH WT, subclass mesenchymal (score 0.4)	PXA (score 0.92)	NA	24	A
11	46	RH	HGG	NA	NA	NA	8	33	D

m: months; RH: right hemisphere; PCF: posterior cranial fossa; SC: spinal cord; LH: left hemisphere; NA: not applicable; PFS: progression-free survival; OS: overall survival; status D/A: Dead/Alive; IHG: infant hemispheric glioma; WT: wild-type; ITD: internal tandem duplication; HGG: high-grade glioma; GBM: glioblastoma; HGGNT: high-grade glioneuronal tumor; HGNET: high-grade neuroepithelial tumor; PXA: anaplastic pleomorphic xantoastrocytoma; NM: no Match.

**Table 2:** First and second-line treatments.

No.	Surgery	1st Line Treatment	Relapse	Progression	Time to Progress (m)	Second Surgery	2nd Line Treatment	Target Therapy	Status (D/A)	OS (m)
1	STR	AIEOP CNS Infants	N	Y	5	N	Y	Y (larotrectinib)	A	47
2	GTR	AIEOP CNS Infants	N	N	NA	N	N	N	A	18
3	GTR	AIEOP CNS Infants	Y	N	31	GTR	N	N	D	64
4	GTR	AIEOP CNS Infants	Y	N	3	GTR	Local RT, bevacizumab and irinotecan every 2 weeks	N	D	96
5	GTR	AIEOP CNS Infants	N	N	NA	N	N	N	A	3
6	STR	AIEOP CNS Infants	N	N	NA	N	N	N	A	3
7	STR	AIEOP CNS Infants	N	Y (RD)	NA	N	Y	Y (larotrectinib)	A	8
8	GTR	AIEOP CNS Infants	Y	N	22	GTR	Local RT, bevacizumab and irinotecan every 2 weeks	N	A	162
9	STR	AIEOP CNS Infants	N	Y	3	GTR	N	N	D	6
10	GTR	RT with TMZ	N	N	NA	N	N	N	A	24
11	STR	RT with TMZ	N	Y	8	N	Bevacizumab and irinotecan every 2 weeks	N	D	33

STR: subtotal resection; NTR: near total resection; GTR: gross total resection; Y: yes; N: no; Status D/A: dead/alive; AIEOP CNS Infants: chemotherapy according to AIEOP guidelines for CNS tumors in infants; RD: refractory disease; RT: radiotherapy; TMZ: temozolomide; Status: D/A: dead/alive; m: months.
